# Enhanced Photocurrent and Electrically Pumped Quantum Dot Emission from Single Plasmonic Nanoantennas

**DOI:** 10.1021/acsnano.3c10092

**Published:** 2024-01-12

**Authors:** Junyang Huang, Shu Hu, Dean Kos, Yuling Xiong, Lukas A. Jakob, Ana Sánchez-Iglesias, Chenyang Guo, Luis M. Liz-Marzán, Jeremy J. Baumberg

**Affiliations:** † NanoPhotonics Centre, Cavendish Laboratory, Department of Physics, JJ Thompson Avenue, University of Cambridge, Cambridge, CB3 0HE, U.K.; ‡ CIC biomaGUNE, Basque Research and Technology Alliance (BRTA), Paseo de Miramón 194, Donostia-San Sebastián 20014, Spain; § Ikerbasque, Basque Foundation for Science, Bilbao 43009, Spain

**Keywords:** plasmonics, nanoantenna, quantum dot, photocurrent, electroluminescence, Stark shift

## Abstract

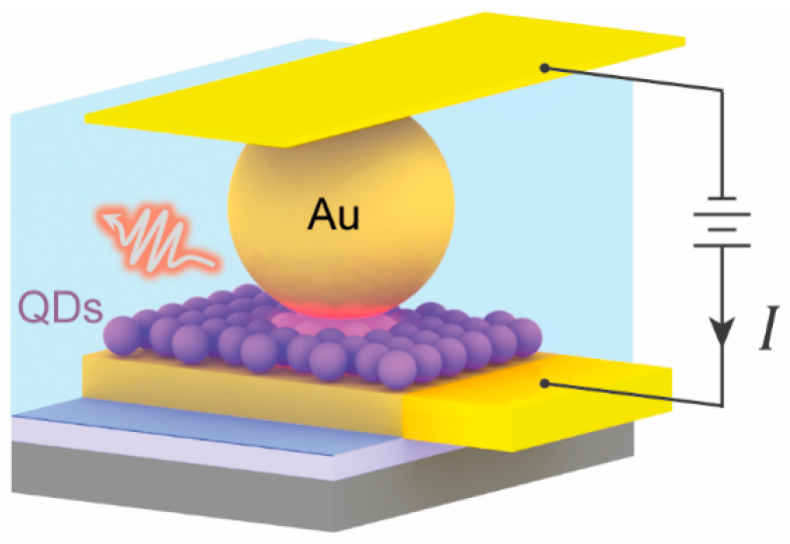

Integrating cavity-enhanced colloidal quantum dots (QDs) into photonic chip devices would be transformative for advancing room-temperature optoelectronic and quantum photonic technologies. However, issues with efficiency, stability, and cost remain formidable challenges to reach the single antenna limit. Here, we present a bottom-up approach that delivers single QD-plasmonic nanoantennas with electrical addressability. These QD nanojunctions exhibit robust photoresponse characteristics, with plasmonically enhanced photocurrent spectra matching the QD solution absorption. We demonstrate electroluminescence from individual plasmonic nanoantennas, extending the device lifetime beyond 40 min by utilizing a 3 nm electron-blocking polymer layer. In addition, we reveal a giant voltage-dependent redshift of up to 62 meV due to the quantum-confined Stark effect and determine the exciton polarizability of the CdSe QD monolayer to be 4 × 10^–5^ meV/(kV/cm)^2^. These developments provide a foundation for accessing scalable quantum light sources and high-speed, tunable optoelectronic systems operating under ambient conditions.

## Introduction

Simple, cost-effective integration of colloidal semiconductor quantum emitters onto chip-based devices has gained substantial momentum owing to its potential to revolutionize optoelectronic and quantum photonic technologies.^1−6^ Serving as both efficient light harvesting agents and solid-state emitters, quantum dots (QDs) have emerged as a pivotal nanomaterial with desirable capabilities, including exceptional tunability of optical and electrical properties, high quantum yield, and flexible compatibility with on-chip integration. Nonetheless, their low spontaneous emission rate (∼100 MHz) and rapid dephasing hinder high-speed quantum photonic applications. Various photonic structures have been devised to overcome these challenges by manipulating the photonic density of states surrounding the QDs, including microcavities,^7,8^ Bragg gratings,^9,10^ whispering gallery mode resonators,^11,12^ photonic crystals,^13,14^ and plasmonic nanocavities.^15−17^

Plasmonic nanocavities have drawn broad attention, since they exhibit compatible quality factors (*Q*) with colloidal QDs at room temperature. Large field enhancements (*E*/*E*_0_ ∼ 100) can be produced in a small optical mode volume (*V* < 10 nm^3^),^18^ enabling strong plasmon–exciton coupling,^19−22^ Purcell enhanced photodetection,^23−25^ brighter QD photoluminescence (∼10^3^-fold), and enhanced emission rates (∼10^2^-fold).^15,26−28^ Although optically pumped QD-plasmonic nanocavity constructs have been studied extensively, robust electrically pumped individual QD-plasmonic nanoantenna devices that are critical for scalable integration within photonic circuit platforms have not yet been achieved.

Electrically contacting single plasmonic constructs with QD integration poses a multitude of challenges, including careful preservation of QD functionality during the optoelectronic device fabrication process (lithography) and spatial alignment of QDs with a few nanometer precision to optimize the coupling efficiency. In addition, the high field confinement and localized surface plasmons introduce substantial Ohmic losses, hindering the overall efficiency of the system. Furthermore, noble metal atoms are known to be mobile under high electric fields at ambient temperatures, leading to the destabilization of the electrical junctions over time.^29^ Therefore, achieving long-term stability and reproducibility necessitates effective control over nanoscale forces and the precise manipulation of chemical interactions surrounding these metal contacts.

Here, we successfully create electrically pumped single QD-plasmonic nanoantennas using a robust bottom-up approach. The method is devoid of electron-beam lithography and leverages liquid–air interface assembly to integrate QDs into nanoparticle-on-mirror (NPoM) plasmonic cavities.^30,31^ Individual plasmonic nanocavities coupled to ∼5 QDs are electrically addressed and demonstrate robust photoresponsive characteristics with wavelength-dependent photocurrent generation matching the QD absorption spectrum. Most notably, we achieve electroluminescence with long-term stability from single nanoantennas through the introduction of an ultrathin electron-blocking polymer layer. The light emission arises from a single NPoM nanocavity dominating electrical transport, revealing quantum-confined Stark effects characterized by a voltage-dependent redshift. These systematic insights into fabrication, electrical properties, and electroluminescent behavior of single QD-plasmonic nanoantennas offer opportunities for the future development of QD-based scalable photonic platforms.

## Results and Discussion

**Figure 1:**
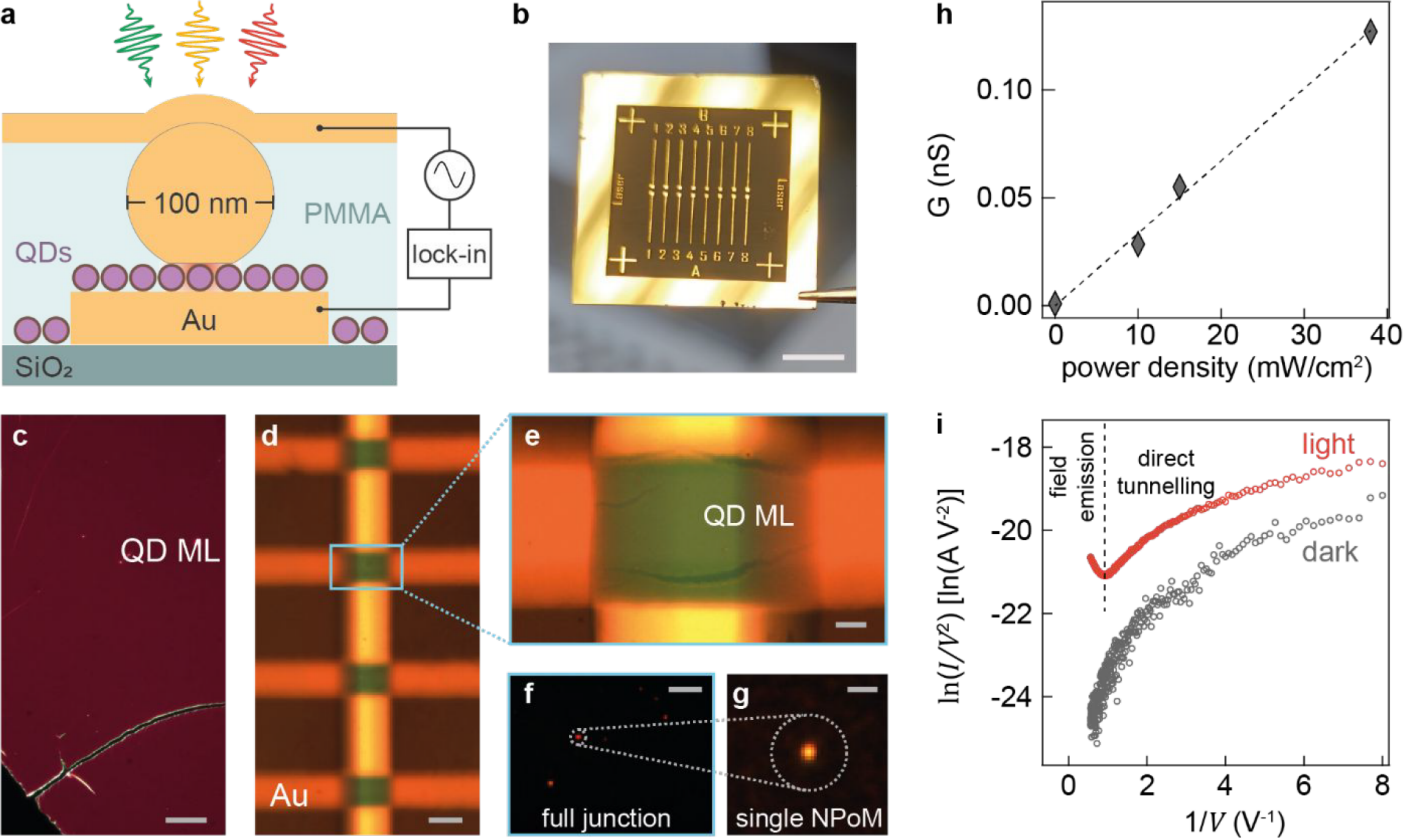
Electrically contacted device with monolayer QDs in a NanoParticle-on-Mirror (NPoM) plasmonic junction. (a) Monolayer of CdSe/CdS QDs sandwiched between a bottom Au mirror electrode and a AuNP, in contact with a semitransparent top gold electrode. (b) Bottom electrodes evaporated on a glass substrate. (c) Dark-field image of a QD monolayer deposited on a Si substrate (scale bar: 20 μm). (d) Upper (horizontal) and lower (vertical) electrodes intersect to form individual cross-bar contact junctions (scale bar: 50 μm). (e) Bright-field image of a cross-bar junction, showing contrast between regions with and without a monolayer of QDs. (f, g) Dark-field scattering of 100 nm diameter NPoM nanoantennas embedded in a cross-bar junction (scale bars: 10 μm in e and 1 μm in f). (h) Conductance in the low-bias region (|*V*| < 1 V) vs illumination intensity with a linear fit (dashed line). (i) Fowler–Nordheim plot of a single QD-NPoM junction with (red) and without (gray) halogen light illumination of 38 mW/cm^2^.

To consistently integrate QDs into NPoMs and create the electrically pumped plasmonic junction (Figure 1a–e), we first employ a liquid–air interface assembly method^30^ to transfer a compact monolayer of CdSe/CdS QDs onto bottom Au electrodes patterned on borosilicate glass. Subsequent sparse deposition of 100 nm AuNPs onto the QD layer forms plasmonic junctions that are further embedded within a spin-coated insulating poly(methyl methacrylate) (PMMA) overlayer. The tops of the AuNPs are then exposed with O_2_ plasma etching, and semitransparent top electrodes of 12 nm Au are thermally evaporated through a customized shadow mask, forming upper lines perpendicular to their lower counterparts. This patterning forms arrays of 320 cross-bar devices per sample, each with an area of 2500 μm^2^ containing 1–10 NPoM junctions (Figure 1f). Each device is individually addressed electrically by contacting the corresponding top–bottom electrode pair using two external probes. Optically isolated NPoM antennas are identified using dark-field microscopy and individually addressed by laser illumination and spectroscopy (Figure 1g).

In this NPoM electrical contact geometry, the bottom facet of the AuNP defines the contact area of the electrical junction. For a typical 100 nm rhombicuboctahedral-type AuNP, this area varies from 700 nm^2^ for triangular facets to 1600 nm^2^ for square facets,^32^ thus covering at most 8 ± 3 hexagonally packed oleic-acid-capped QDs (12 nm in diameter) in a monolayer junction. Measuring the dark-field spectral resonance of >2600 bare QD NPoM nanocavities yields a narrow distribution of peak wavelengths with a full width at half-maximum (fwhm) of 50 nm (Figure S1). These nanocavity resonances are highly sensitive to the gap geometry, and since this fwhm matches that of NPoMs with gaps defined by self-assembled molecular monolayers,^33^ it suggests the variation is mainly from the size inhomogeneity of AuNPs and bottom facet shape.^32^ Such gap uniformity is also verified with AFM imaging of the QD monolayer on Au (Figure S2).

The current–voltage (*I*–*V*) characteristics of the NPoM junction show that they are photoresponsive for different illumination conditions. Both in the dark and with white illumination, symmetric *I*–*V* characteristics are observed for both forward and reverse bias (Figure S3a). This symmetry is attributed to the inherently symmetric metal–QD–metal junction, which benefits device stability and simplifies AC operation. Higher current under illumination is attributed to photoinduced charge separation and extraction from the QDs, with dark current levels of <100 pA. The conductance values (*G*) of the NPoM extracted from linear fits of the Ohmic region (±1 V) show linear dependence on incident optical power density (Figure 1h).

The Fowler–Nordheim (F–N) model, *I* ∝ *V* exp(−κ/*V*), is applied to confirm the charge transport mechanism of the QD junction (Figure 1i), where κ is a constant associated with the tunneling barrier formed at the heterojunction boundary under high electric fields *V*.^34,35^ Light-generated excitons in the QDs dissociate to give an additional photocurrent. The *I*–*V* characteristics in the F–N coordinates exhibit a positive gradient in the low-bias region (|*V*|^–1^ > 1.0 ± 0.1 V^–1^), implying charge transport is dominated by direct tunneling through barriers of the CdS shell and QD ligands. At higher bias voltages (|*V*|^–1^ < 1.0 ± 0.1 V^–1^) a negative slope is observed with light, evidencing field emission through triangular barriers (Figure 1i, red). In the dark, however, the transition from direct tunneling to field emission is not observed (Figure 1i, gray), confirming that charge injection from the metal into the QDs is also governed by direct tunneling.

**Figure 2:**
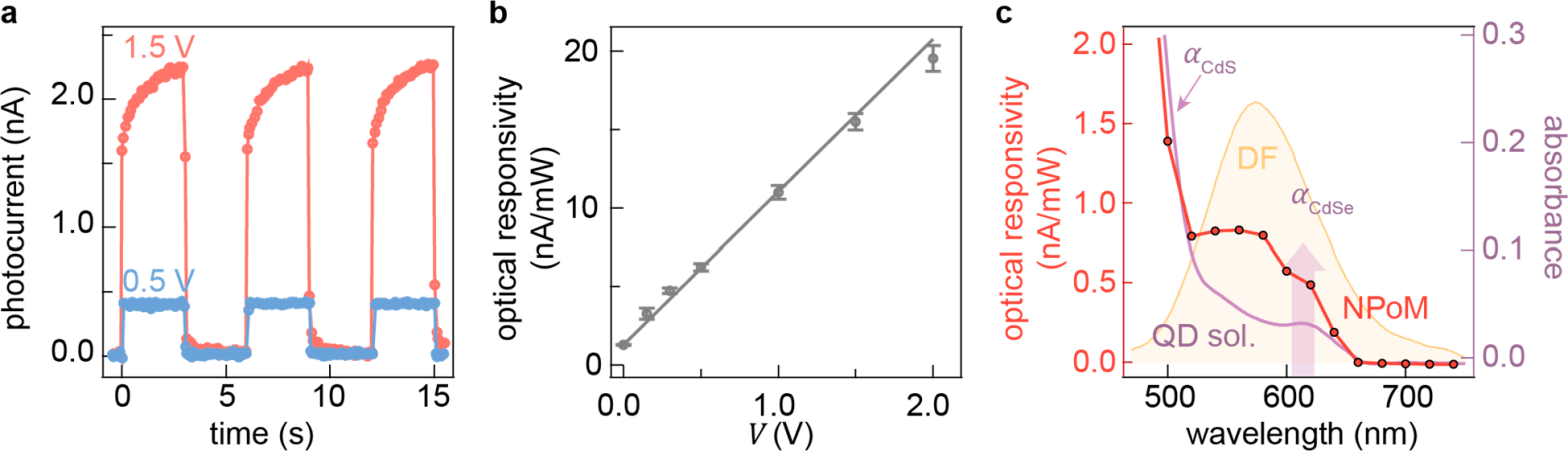
Photocurrent generation in QD NPoM devices. (a) Photocurrent time trace measured on an NPoM device with bias voltages of 0.5 (blue) and 1.5 V (red). Incident white halogen illumination of 38 mW/cm^2^ with 6 s modulation. (b) Optical responsivity from a single NPoM junction under 633 nm laser illumination as a function of bias voltage, with linear fit. Average optical power and modulation amplitude are 150 and 50 μW, respectively. (c) Responsivity versus optical excitation wavelength (red, *V* = 0.5 V, *I*_power_ = 40 μW) plotted with the extinction spectrum of CdSe/CdS QDs measured in hexane solution (purple) and dark-field scattering profile of the same single QD NPoM device (yellow). Arrows mark exciton absorption resonances for CdSe and CdS. Responsivities in (b, c) are taken after the photocurrent saturates to its final value.

The real-time current response of each NPoM junction is investigated under large-area white light illumination with a 6 s modulation period at a fixed bias (Figure 2a, Figure S3). When biased in the direct tunneling regime (*V* = 0.5 V), a stable photocurrent is recorded under illumination with a fast turn-on and turn-off response (<100 ms). Different photocurrent dynamics are captured in the field emission regime (*V* = 1.5 V) with a slow continuous rise (τ_rise_ = 0.9 ± 0.1 s) of photocurrent during illumination, accompanied by a more rapid decay when the light is switched off (τ_decay_ = 0.3 ± 0.1 s). This phenomenon is attributed to the influence of the electric field strength on the QD photocurrent close to the NPoM junction. Specifically, in low electric fields, the photocurrent is primarily governed by QDs situated in the immediate vicinity of the junction, whereas a higher electric field promotes charge extraction from participating QDs distributed further across the monolayer. The QDs located farther from the plasmonic junction experience longer travel paths to reach the NPoM, enhancing the likelihood of carrier trapping at defects in the QD film and giving rise to the slower photocurrent kinetics.^36^

To perform optical responsivity measurement for individual NPoM junctions, we intensity-modulate a diffraction-limited 633 nm laser focused on each structure and detect the photocurrent using lock-in amplification. Scanning the laser spot across different spatial locations confirms that only a single NPoM junction produces photocurrent activity from the device despite the presence of several other NPoM structures within the cross-bar area. This indicates each device is dominated by a single NPoM junction that shorts out lower conductivity junctions, in agreement with observations on self-assembled molecular NPoM devices.^37^ Varying the bias voltage from 0 to 2 V while keeping the average optical power at 150 μW and modulation amplitude at 50 μW (for lock-in detection), a linear increase in optical responsivity with applied bias voltage is observed (Figure 2b). Within this bias range below the band edge exciton energy (<2 eV), the device maintains a stable current level (Figure S3e). The observed linear response suggests that Schottky tunneling through the ultrathin barrier may not be the rate-determining factor, and the separation and collection of optically excited electron–hole pairs can be modulated by trapped photoinduced charge carriers around the QDs. A similar linear dependence of photoresponsivity with bias voltage has also been identified in MoS_2_ heterojunctions.^38,39^

We further assess the wavelength dependence of photocurrent generation, exciting individual NPoM junctions with a spectrally filtered supercontinuum laser. The optical power is kept constant at 40 μW as the spectral window of 20 nm bandwidth is varied from 480 to 740 nm. The optical responsivity spectrum is in good agreement with the UV–vis spectrum of QDs in solution with QD excitonic absorption features resolved for both CdSe and CdS (arrows in Figure 2c). The optical responsivity is further enhanced in the region between 550 and 640 nm, coinciding well with the dark-field scattering spectrum of the same NPoM, thus evidencing plasmonic enhancement of the photocurrent.

**Figure 3:**
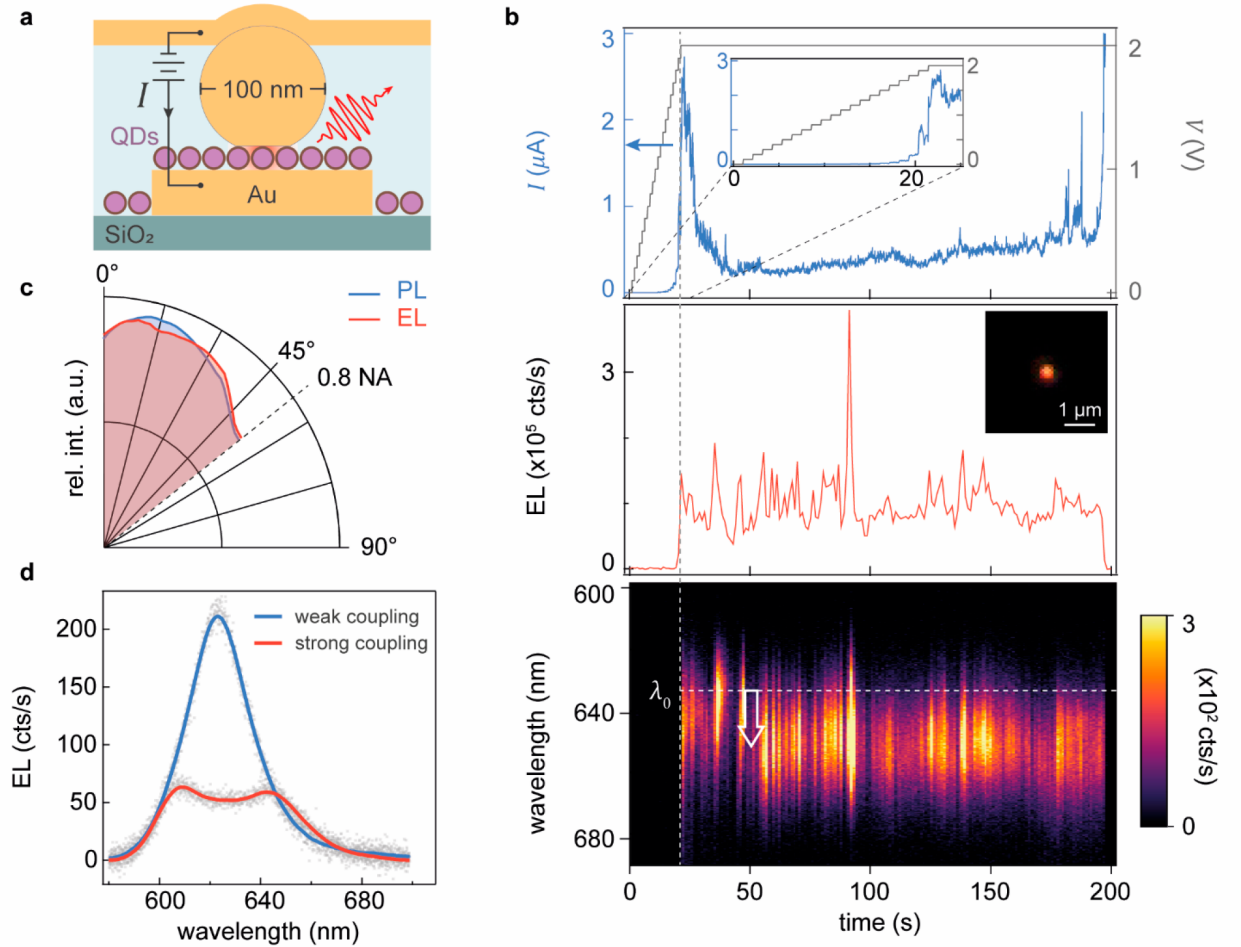
Electroluminescence of the QD-NPoM device. (a) Schematic of single-junction electroluminescence (EL) experiment. (b) Top panel: bias voltage (gray) and current (blue) vs time during electrical pumping of a single QD NPoM device; middle panel: time trace of total EL intensity and EL image (inset); bottom panel: time evolution of QD EL spectrum. Maximum EL count rates exceed 400 kcts/s. (c) Experimental angular emission pattern using optical (blue) and electrical (red) excitation, with 0° normal to the sample plane. (d) EL spectra of a nanoantenna-dressed QD in weak (blue) and strong (red) coupling regimes.

Thorough characterization of the optoelectronic response allows the exploration of electrically pumped emission from such QD-NPoM devices (Figure 3a). We first identify the active NPoM within a cross-bar junction by mapping the photocurrent response under low DC bias (<0.5 V). Subsequently increasing the DC bias across the nanogap to ∼2 V elicits electroluminescence (EL) originating from a diffraction-limited spot, giving integrated count rates of >10^5^ cts/s (Figure 3b). This electrical activation threshold agrees well with the first exciton energy of CdSe QDs. A rapid surge in current intensity is observed upon initiation of the EL, attributable to charge stabilization processes within the ligand layer or ligand rearrangement, followed by a return to an average current of ∼0.5 μA. While fluctuations manifest in both current and EL intensity, no discernible correlation is found between them, suggesting they arise from different mechanisms. While EL depends on local field fluctuations throughout the QD from moving defects and charges, current fluctuations primarily are controlled by instabilities in the contact barriers near the interfaces.

Despite the simplicity of this design, these metal–QD–metal junctions demonstrate robust performance under DC electrical pumping, sustaining emission for durations of over 3 min. Their vulnerability arises from the migration of surface atoms on the Au facet that ultimately short-circuits the device. To address this issue, we introduce a thin electron-blocking polymer layer beneath the AuNP facet to stabilize the nanogap on the upper Au surface. Integrating such a sub-5-nm charge-blocking layer within the confined plasmonic nanogap is challenging. We achieve this by enveloping the AuNP with PEDOT:PSS before selectively preserving the polymer layer within the gap during plasma etching. This intervention successfully extends the device lifetime by 1000-fold (Figures S4, S5), underscoring its potential for practical application. The slow temporal evolution of the EL spectrum (lower panel Figure 3b) shows spectral wandering and intensity flickering in time, indicating that instabilities in the local environment cause fluctuations in electric field strength and electrical tunnelling conditions. Tuning the optimal coupling to the QD band-edge exciton in these plasmonic cavities, we observe Rabi splitting in the EL spectrum (Figure 3d), explored in detail elsewhere.^31^

The angular emission profile of these devices is recorded for both PL and EL. In both cases, the NPoM devices emit in a broad angular range spanning from 0° to 55° (Figure 3c). To confirm that the device can provide single-photon emission, photon correlation experiments were developed; however the typical Purcell enhancement in such plasmonic nanocavities is predicted to reduce the radiative lifetime by ∼10^3^-fold.^15^ This effect is anticipated to be significant in the NPoM geometry due to the strong field confinement.^31^ Given the bare QD lifetime τ_0_ ∼ 30 ns,^40^ we estimate cavity-enhanced lifetimes to be <10 ps, which is far below the resolution limit (1 ns) of our single-photon detectors, preventing so far the effective characterization of *g*^(2)^ correlations in EL (Figure S6).

### Stark Shift in Electroluminescence

**Figure 4:**
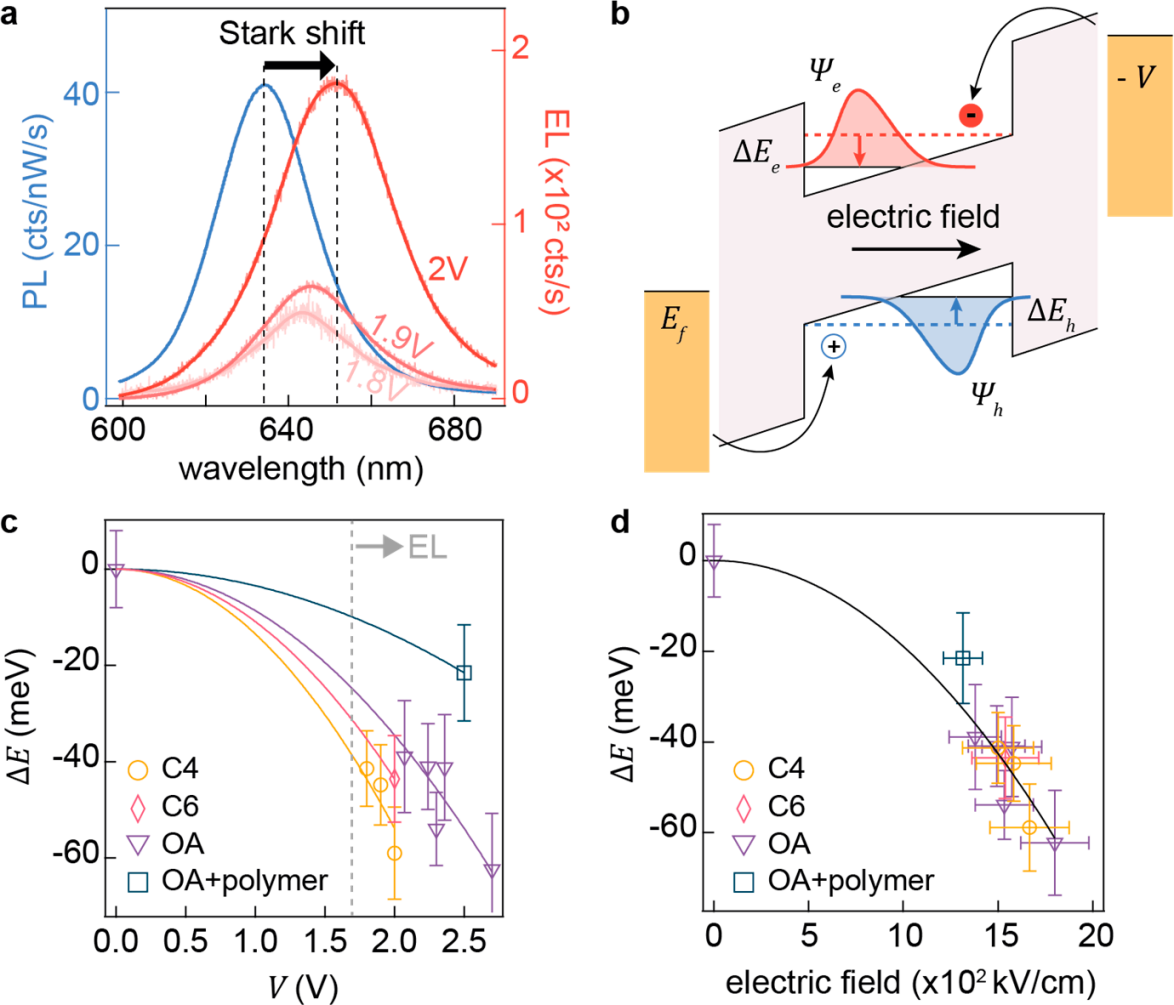
Stark shift in electroluminescence. (a) Stark-shifted EL spectra at increasing bias voltages (red) compared to the photoluminescence spectrum at *V* = 0 (blue) from the QD NPoM device. (b) Schematic of QD quantum-confined Stark effect under a DC electrical field between metal contacts. Stark shift versus (c) bias voltage and (d) calculated electric field for gap sizes defined by different ligands around the QD monolayer; data points fit with parabolic functions.

In EL, the exciton energy is found to exhibit a voltage-dependent redshift (Δλ ∼ 20 nm at 2 V) with respect to the PL peak wavelength measured without electrical bias (Figure 4a, statistical distribution in Figure S7), accompanied by a nonlinear rise in the EL intensity with increasing bias. Such energy shifts are a result of the quantum-confined Stark effect (QCSE).^41−43^ Under DC bias, the internal electric field induced inside the QD drives the tunnel-injected electron and hole in opposite directions, giving rise to reduction of the band gap that induces a redshift in EL (Figure 4b).^44^ The resulting energy shift is given by Δ*E* = −*μF* – *αF*^2^, where *F* is the electric field and μ and α are the components of permanent dipole moment and the polarizability in the direction of the electric field. Owing to its spherical symmetry, the permanent dipole moment in these QDs is negligible (μ ≃ 0), and therefore the QCSE is expected to have a purely quadratic field dependence.

We thus further adjust the NPoM gap size by dressing QDs with ligands of various lengths, which include butyric acid (C4, 0.5 nm), caproic acid (C6, 1 nm), and oleic acid (OA, 2 nm), as well as employing thicker polymer spacing. A quadratic trend is indeed observed under varying EL biases for this range of NPoM gap variations (Figure 4c). As the gap size expands, a larger EL voltage threshold is observed, accompanied by a reduced sensitivity in Stark tuning. Converting the bias into electric fields using the estimated ligand lengths, the Stark tuning observed in EL across a variety of NPoM gaps falls onto a single quadratic function of electric field (Figure 4d). By fitting the experimental data, we obtain a value of α = 4 × 10^–5^ meV/(kV/cm)^2^ for the exciton polarizability of a CdSe QD monolayer. This value is within ∼50% of literature findings for field-dependent PL measurements conducted on bulk CdSe films^45,46^ and compares to theoretical estimates for the polarizability of a CdSe exciton at λ_PL_ = 637 nm of 1.3 × 10^–4^ meV/(kV/cm)^2^.^47−49^ Discrepancies between measured and calculated values suggest that the field perturbation experienced by the CdSe core may be reduced by charge-trapping sites in QDs, screening effects from the nearby metal, and surface roughness caused by atomic migration. We note that the root-mean-square optical field in the nanogap under white illumination is on the order of *E*_rms_ ∼ 10 V/cm, which is 10^5^ smaller than the DC field strength, and thus negligible. The Stark shift of the electroluminescence here is comparable with its room-temperature spectral line width, thereby affording prospects for wavelength manipulation in the development of future chip-based photon sources.

## Conclusions

In conclusion, we demonstrate electrically driven QD-plasmonic nanoantennas fabricated through a colloidal-based bottom-up fabrication technique. We reveal a robust photoresponsive behavior from QD integrated plasmonic junctions and obtain electroluminescence from individual nanoantennas over extended periods. This compact nanogap geometry induces significant quantum-confined Stark shifts in QD electroluminescence, exceeding 60 meV at 2 V. Further focus on enhancing stability and photon collection efficiency holds the potential to optimize this CMOS-compatible fabrication approach, offering routes to cost-effective and scalable integration of on-chip light sources.

The accelerated emission rate of these QDs presents an exciting prospect for interfacing with fast electronics at clock speeds exceeding 10 GHz. Crucially, the versatility of this technique extends its applicability to diverse quantum emitters spanning visible and NIR wavelengths, facilitating the development of large-scale quantum light sources in integrated photonics and high-speed, wavelength-tunable optoelectronic devices. Moreover, our system represents an intriguing platform for future nanoantenna-enhanced charge transfer studies, providing opportunities to understand and manipulate catalysis, electrochemistry, and photovoltaics at the nanoscale.

## Materials and Methods

### QD Monolayer Assembly

Colloidal CdSe/CdS quantum dot solutions in hexane (λ_abs_ = 627 nm, λ_emi_ = 637 nm, *D*_QD_ = 11.2 ± 0.8 nm) were purchased from Fraunhofer CAN with a concentration of 10 mg·mL^–1^. In a typical assembly process for a QD monolayer, the QD solution is diluted 50 times with hexane before spreading 100 μL of solution on a diethylene glycol (DEG) surface in a clean Petri dish. Covering the Petri dish allows hexane to slowly evaporate over 5–10 min and form a compact monolayer film on the DEG–air interface.^30^ To transfer the QD film, the glass substrate is first hydrophobized with 1:1000 (3-aminopropyl)triethoxysilane solution before being gently brought into contact with the QD film. Lifting up the substrate transfers centimeter-scale QD monolayers, which are then washed with 99% ethanol and blow dried with nitrogen. The thickness of the QD monolayer is characterized to be 16 ± 3 nm using atomic force microscopy (Figure S2).

### Device Fabrication

Electrical contact devices are fabricated on borosilicate glass substrates (Pi-Kem).^37^ A Cr adhesion layer (3 nm) and Au bottom electrode (30 nm) are first deposited on the glass substrate via thermal evaporation at a rate of 0.1 nm/s (NanoPVD-T15A, Moorfield Nanotechnology) through shadow masks (PhotoFab, Alphasol Tec AG). The monolayer QD film is then transferred onto the bottom electrode with AuNPs (BBI solutions, *D* = 100 nm) sparsely drop-cast and washed with deionized water afterward. Solution concentration and deposition time are controlled such that only 1–10 NP junctions are created within each cross-bar device. PMMA (molecular weight, 950 kg/mol) at 2 wt % in anisole (MicroChem) is spin coated at 2 krpm and baked on a 50 °C hot plate for 2 min to yield a final thickness of 100 nm above the surface of the bottom electrode. Oxygen plasma (HPT-100, Henniker Plasma) is used to etch away 20 nm of the PMMA layer, exposing the top of the AuNPs. Lastly, top electrodes of 12 nm Au are thermally evaporated through a customized shadow mask with electrode patterns perpendicular to their bottom counterpart. To create the PEDOT:PSS polymer coating around AuNPs, 30 to 80 μL of PEDOT:PSS aqueous solution (3–4%, Sigma-Aldrich) is mixed with 1.6 mL of a 100 nm AuNP suspension (BBI Solutions) and incubated at 4 °C for 15 h. Supernatant and excess polymer are replaced with deionized water after centrifugation. To form the NPoM structure used in Figure S5c,d, PEDOT:PSS core–shell AuNPs are drop-casted on a template-stripped Au film before being blow-dried with nitrogen gas.

### Optical Measurement

Dark-field microscopy and spectroscopy are performed with a customized Olympus BX51 microscope. Dark-field scattering is recorded on a confocal fiber-coupled spectrometer (QE65000, Ocean Optics) with 1.5 μm collection spot diameter on the sample. EL and PL signals are collected using a 100× Olympus objective (0.8 NA) coupled through a Triax 320 spectrometer and recorded on an Andor Newton EMCCD using an integration time of 1 s. Optical excitation of NPoMs is realized by focusing a spectrally filtered CW diode laser at 447 (Coherent CUBE) or 633 nm (Integrated Optics) through the same objective. Fourier space microscopy for QD emission is achieved by capturing the emission pattern from an NPoM device at the back focal plane (BFP) of the objective and relaying the BFP image to an Andor Newton 970 BVF EMCCD.

### Electrical Measurement

Electrodes on the device are contacted via tungsten probes (American Probe & Technologies) mounted in a customized probe station integrated into the microscope stage. DC bias and *I*–*V* curves are performed with a source-measure unit (2635A, Keithley). For monochrome photocurrent measurement, the 633 nm CW laser is modulated at 1 kHz with an acousto-optic modulator (AOM). The AOM modulation signal is synced to a lock-in amplifier (SR810, Stanford Research Systems) in first-harmonic current detection mode for measuring the photocurrent generated from the device. Photocurrent spectroscopy is realized using a filtered supercontinuum source (Fianium fiber laser, SuperChrome filter unit) with 20 nm fwhm bandwidth, modulated at 788 Hz by a chopper. The optical excitation power on the sample is calibrated using a power meter and a motorized continuous neutral density filter wheel, thus kept constant at all wavelengths. All measurements are conducted at ambient conditions.

## Data Availability

All data needed to evaluate the conclusions in the paper are present in the paper and/or the Supporting Information.

## Supporting Information Available

Histogram of QD NPoM cavity resonances; AFM data for QD monolayer roughness; additional *I*–*V* characterization and photocurrent measurement; characterization of AuNP@PEDOT:PSS core–shell particles and NPoM EL device with PEDOT:PSS layer; photon coincidence correlation measurements and statistical distribution of EL peak wavelength (PDF)
