# Supplementary material for: Enhanced Photocurrent and Electrically Pumped Quantum Dot Emission from Single Plasmonic Nanoantennas
Source: ACS Nano. 2024 Jan 12;18(4):3323–30. doi: 10.1021/acsnano.3c10092 (PMC10832344; doi:10.1021/acsnano.3c10092)
Supplement: Supplementary file 1 — nn3c10092_si_001.pdf [file nn3c10092_si_001.pdf]

Supporting Information for

Enhanced Photocurrent and Electrically-pumped Quantum Dot Emission from Single Plasmonic Nanoantennas

Junyang Huang[1†], Shu Hu[1†], Dean Kos[1], Yuling Xiong[1], Lukas A. Jakob[1], Ana Sánchez-Iglesias[2], Chenyang Guo,[1] Luis M. Liz-Marzán[2,3*], Jeremy J. Baumberg[1*]

[1]NanoPhotonics Centre, Cavendish Laboratory, Department of Physics, JJ Thompson Avenue, University of Cambridge, Cambridge, CB3 0HE, UK

[2] CIC biomaGUNE, Basque Research and Technology Alliance (BRTA), Paseo de Miramón 194, Donostia-San Sebastián 20014, Spain

[3] Ikerbasque, Basque Foundation for Science, Bilbao 43009, Spain

[†] These authors contributed equally to this work

[*]Corresponding authors. Email: jjb12@cam.ac.uk (J.J.B.), llizmarzan@cicbiomagune.es (L.L-M.)

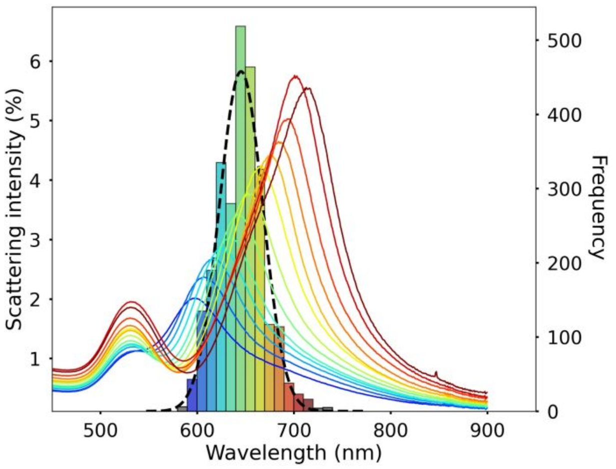

Fig. S1. Histogram of the dominant cavity mode wavelengths, along with average dark-field spectra from each bin of 100 nm QDs integrated into NPoMs.

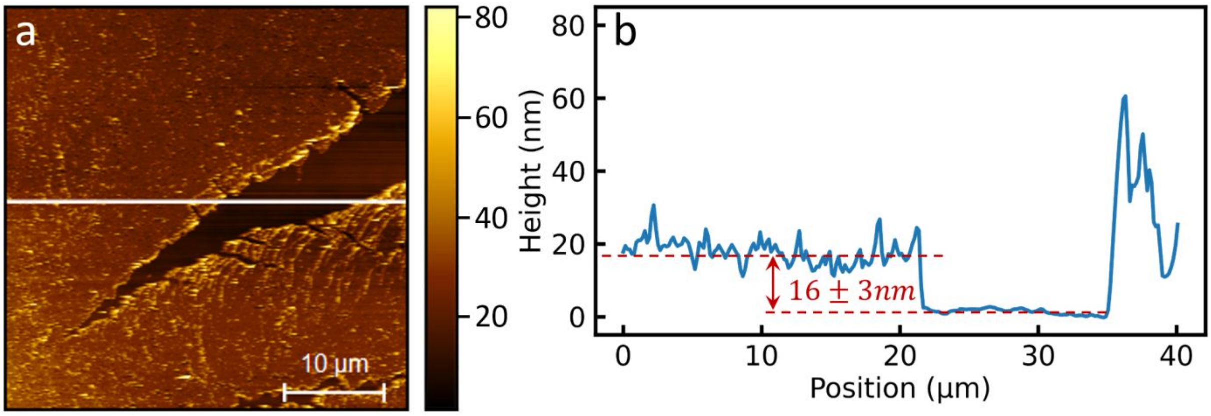

Fig.S2. Atomic force microscopy (a) image and (b) linescan at edge of the monolayer QDs film for extracting the height of the QDs.

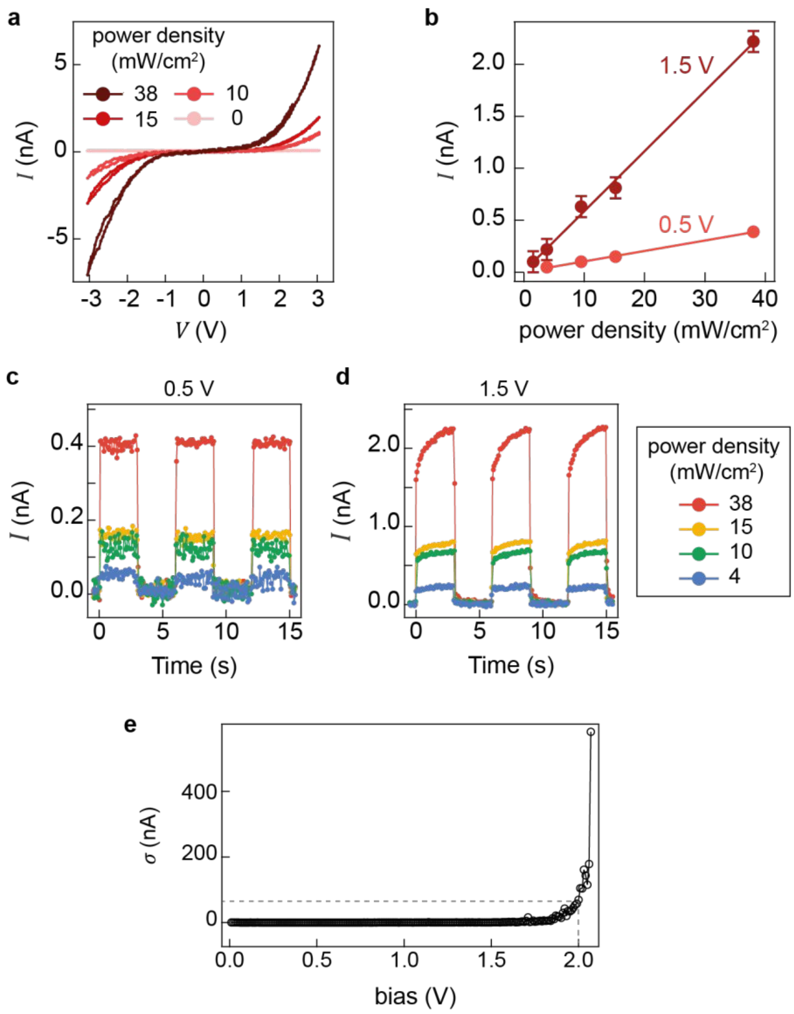

Fig. S3. I-V characterization and photocurrent measurement. (a) I-V curve of crossbar junction under halogen lamp illumination at various power densities. (b) Photocurrent as a function of power density of illumination under 0.5 V and 1.5 V bias. Photocurrent time trace of an NPoM device various halogen illumination intensities (6s cycle time), under bias voltage (c) V = 0.5 V and (d) 1.5 V. (e) Standard deviation of current noise at increasing bias, showing stability below 2V.

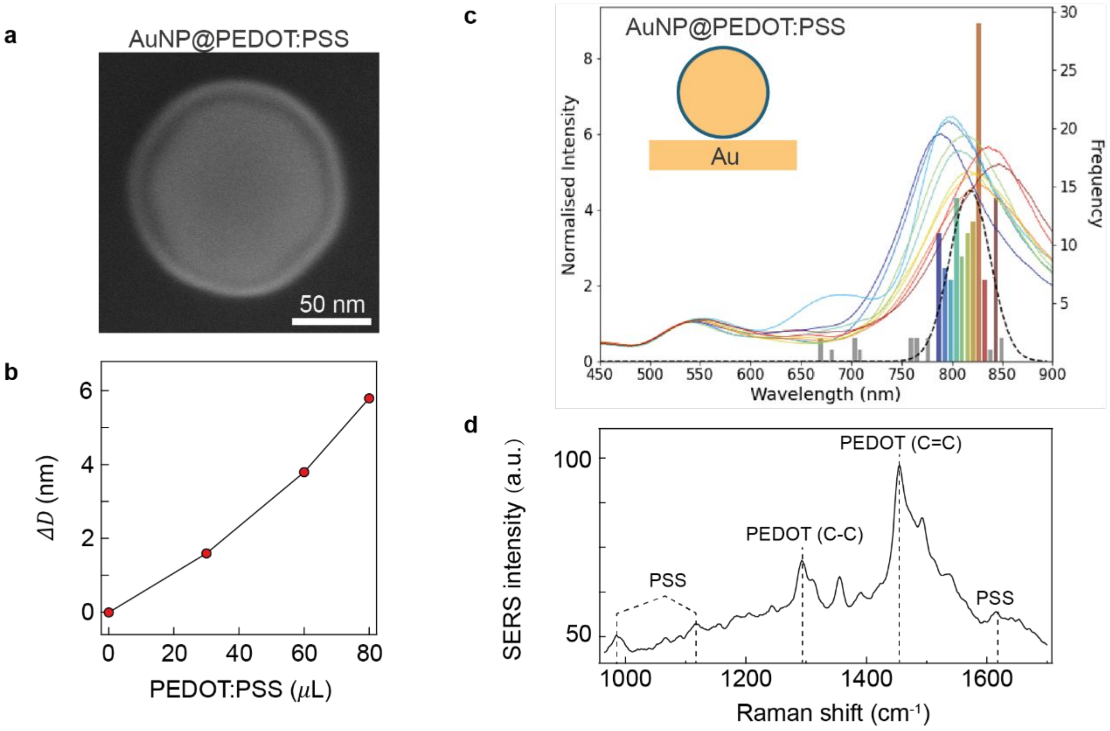

Fig. S4. AuNP@PEDOT:PSS coreshell particle. (a) SEM image shows the core-shell structure of the PEDOT:PSS coated AuNP. (b) Increased polymer coating thickness characterised using dynamic light scattering, as a function of PEDOT:PSS solution volume used in the incubation process. The highest shell thickness (with 80 μL) was used in measurements (c-h). (c) Distribution of PEDOT:PSS coreshell NPoM (inset) dark-field scattering coupled-mode resonances. Averages of spectra in each histogram bin of the corresponding colours exhibit a centre wavelength of 815 nm. (d) Surface-enhanced Raman spectrum of PEDOT:PSS coreshell NPoM (λ_{ext} = 633 nm), showing characteristic vibration signatures PEDOT and PSS.

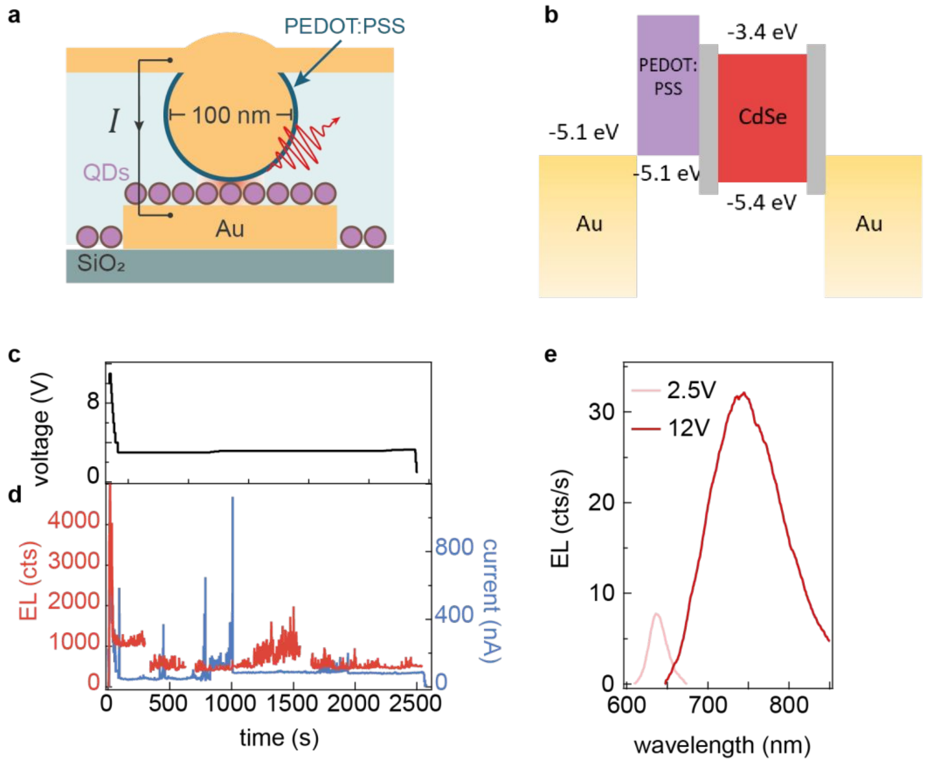

Fig. S5. NPoM device with PEDOT:PSS layer. (a) Schematic illustration for single junction electroluminescence (EL) experiment with PEDOT:PSS coated NPoM, where polymer on top of AuNP was etched away, enabling direct contact with the top electrode. (b) Flat-band energy level diagram of the NPoM device junction. Time traces of (c) bias voltage, (d) current (blue) and EL intensity during electrical pumping of a single PEDOT:PSS-QD NPoM device. The EL intensity was measured using a Lumenera Infinity miscroscope camera. Intermission in the time trace was due to software data processing. (e) EL spectra acquired at 2.5V and 12V.

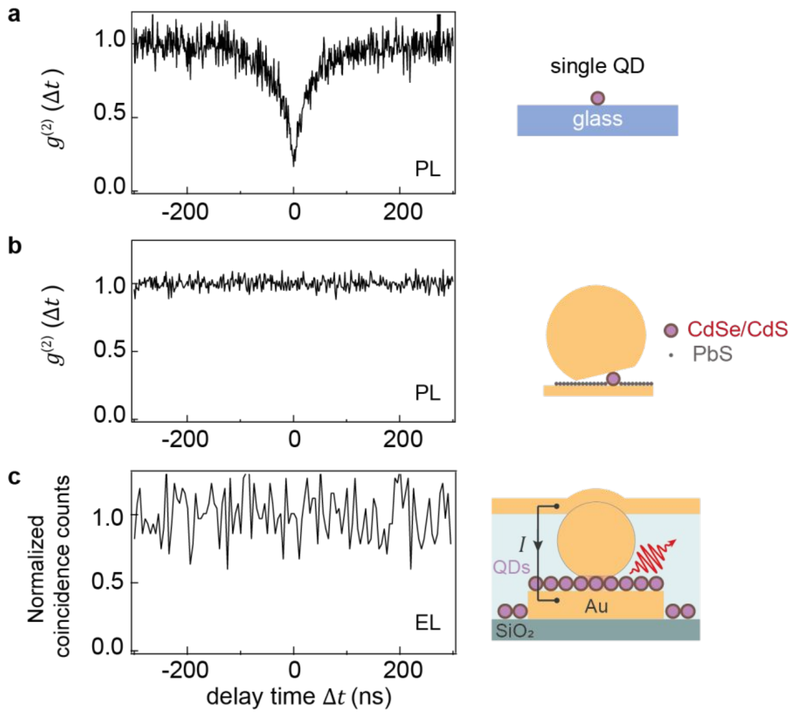

Fig. S6. Second order correlation function measured using avalanche photodiodes (APDs) on (a) single CdSe QDs on glass, and (b) NPoM nanocavity containing a single CdSe QD, under 448 nm continuous wave optical excitation. (c) Normalized coincidence counts as a function of delay time of electroluminescence signal from QD-NPoM device. CdSe QD emission is filtered using a 10 nm bandpass filter before separated by a 50:50 beamsplitter and recorded on a pair of avalanche photo diodes. The average PL count rate is 7200 and 29000 cts/s for bare QD on glass and the QD NPoM, respectively. In EL, the average count rate is 1300 cts/s, with the maximum count rate recorded of 14400 cts/s.

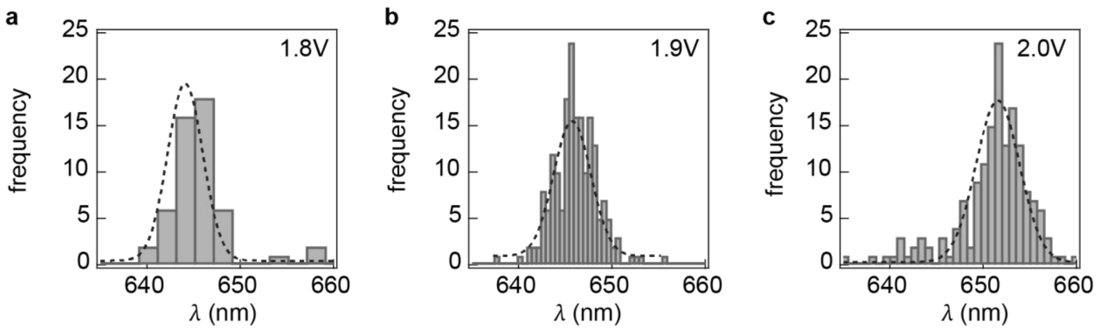

Fig. S7. Bias-dependent electroluminescence. Statistical distribution of EL peak wavelength under bias voltage of (a) 1.8 V, (b) 1.9 V, and (c) 2.0V. Dashed lines represent Gaussian fits of 644 ± 2 nm, 646 ± 3 nm, and 651 ± 3 nm, respectively.
